# Reversible Severe Left Ventricular Dysfunction in an Adolescent With Vitamin D Deficiency-Associated Hypocalcemia and Secondary Hyperparathyroidism: A Case Report

**DOI:** 10.7759/cureus.108534

**Published:** 2026-05-09

**Authors:** Rashmi Patil, Guna Sai Vallapuri, Pankaj Jariwala, Anjani Matturi

**Affiliations:** 1 Cardiology, Yashoda Super Speciality Hospitals, Hyderabad, IND

**Keywords:** adolescent, dilated cardiomyopathy, functional mitral regurgitation, functional tricuspid regurgitation, heart failure, hypocalcemia, hypomagnesemia, reversible cardiomyopathy, secondary hyperparathyroidism, vitamin d deficiency

## Abstract

Hypocalcemia is an uncommon but clinically important and potentially reversible metabolic contributor associated with left ventricular (LV) systolic dysfunction and heart failure. Vitamin D deficiency can precipitate hypocalcemia with secondary hyperparathyroidism, and delayed recognition may lead to severe decompensation. We report the case of a 13-year-old female patient with decompensated heart failure and dilated cardiomyopathy with severe LV dysfunction and severe functional mitral and tricuspid regurgitation. Evaluation revealed profound hypocalcemia with low ionized calcium, persistent hypomagnesemia, low 25-hydroxyvitamin D, and markedly elevated parathyroid hormone (PTH), consistent with a multifactorial metabolic derangement including vitamin D deficiency-associated secondary hyperparathyroidism. She was treated with decongestive therapy and guideline-directed heart failure pharmacotherapy alongside targeted correction of calcium, magnesium, and vitamin D deficiency. Serial echocardiography documented progressive recovery of LV systolic function (ejection fraction (EF) 35% at baseline, improving to 40% at one month and 55% at three months), and echocardiography at the one-year follow-up confirmed normal biventricular function (EF 65%) with resolution of valvular regurgitation. This case highlights that systematic screening for calcium, ionized calcium, magnesium, PTH, and vitamin D should be incorporated early in young patients with severe or disproportionate LV dysfunction because identifying metabolic contributors can alter management and prognosis.

## Introduction

Hypocalcemia-associated cardiomyopathy is rare but clinically high impact because timely recognition and biochemical correction can lead to substantial improvement in systolic function, although it is often multifactorial and may coexist with other reversible or primary myocardial processes. A pediatric review of hypocalcemic rachitic cardiomyopathy emphasizes profound hypocalcemia as a treatable cause of heart failure and highlights recovery when identified early [1]. Adult and pediatric reports describe improvement in left ventricular (LV) function after correction of hypocalcemia and vitamin D deficiency [2,3]. Cardiac magnetic resonance imaging (CMR) may add diagnostic and prognostic value in pediatric hypocalcemic dilated cardiomyopathy, particularly when myocarditis is a competing diagnosis [4]. The present report is notable for an adolescent phenotype with documented ionized hypocalcemia and marked secondary hyperparathyroidism, severe functional mitral/tricuspid regurgitation at presentation, and a serial ejection fraction (EF) recovery trajectory culminating in normalization at the one-year follow-up, supporting a reversible, metabolically mediated cardiomyopathy phenotype.

## Case presentation

A 13-year-old female patient presented with progressive breathlessness for 10 days, with facial puffiness progressing to generalized edema over approximately one month. She had New York Heart Association (NYHA) class III symptoms at presentation [5]. Vital signs were stable (temperature 36.7°C, pulse 88 beats/minute, blood pressure 120/80 mmHg, respiratory rate 20 breaths/minute). Clinical assessment was consistent with congestive heart failure, prompting inpatient evaluation for precipitants and reversible contributors. Given the severity of LV dysfunction and mild troponin elevation, myocarditis and primary dilated cardiomyopathy were also considered; the subsequent clinical course and serial EF recovery supported a likely metabolic contribution with a reversible phenotype, although alternative etiologies could not be fully excluded. 

Diagnostic evaluation

Echocardiography during admission documented dilated cardiomyopathy with severe LV systolic dysfunction and severe mitral and tricuspid regurgitation, interpreted clinically as functional. Laboratory evaluation demonstrated the following findings, as summarized in Table 1.

**Table 1:** Baseline laboratory investigations with age-appropriate reference ranges Laboratory measurements were obtained from blood samples using standard clinical assays (serum, plasma, or whole blood as appropriate). PTH: parathyroid hormone; NT-proBNP: N-terminal pro–B-type natriuretic peptide; CRP: C-reactive protein; TSH: thyroid-stimulating hormone; ANA: antinuclear antibody.

Parameter	Patient Value	Reference Range
Total calcium	6.10 mg/dL	8.8–10.8 mg/dL
Ionized calcium	0.79 mmol/L	1.12–1.32 mmol/L
Magnesium	1.50 mg/dL	1.7–2.2 mg/dL
Phosphate	5.0 mg/dL	3.0–5.5 mg/dL (adolescents)
25-hydroxy vitamin D	16.6 ng/mL	≥20 ng/mL (insufficient <20; optimal ≥30)
PTH	658.5 pg/mL	10–65 pg/mL
NT-proBNP	13,200 pg/mL	<300 pg/mL (children >1 year; assay-dependent)
Troponin-I	0.303 ng/mL	<0.04 ng/mL
Albumin	4.10 g/dL	3.8–5.4 g/dL
CRP	5.0 mg/L	<1 mg/L
TSH	2.44 mIU/mL	0.5–4.3 mIU/mL
ANA Profile	Negative	Negative

The biochemical pattern supported a multifactorial metabolic derangement characterized by vitamin D deficiency, secondary hyperparathyroidism, and hypomagnesemia.

**Figure 1:**
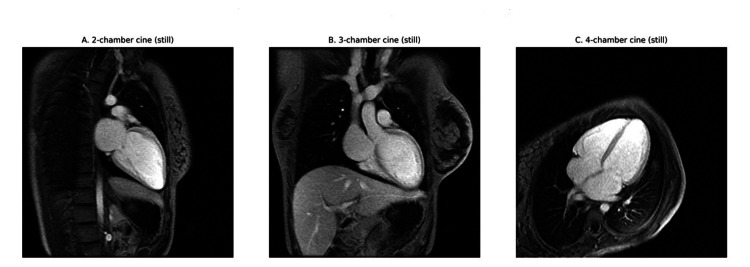
Cardiac magnetic resonance long-axis cine still frames. (A) Two-chamber long-axis view demonstrating a dilated left ventricle and left atrium. (B) Three-chamber long-axis view demonstrating a dilated left ventricle with visualization of the left ventricular outflow tract and aortic root. (C) Four-chamber long-axis view demonstrating dilation of the left ventricle.

CMR cine long-axis imaging (two-chamber, three-chamber, and four-chamber views) demonstrated a dilated LV with markedly reduced systolic function, consistent with severe systolic dysfunction. Tissue characterization demonstrated no myocardial edema and no late gadolinium enhancement, suggesting the absence of active inflammatory injury and replacement fibrosis. CMR long-axis cine still frames are shown in Figure 1.

Management and hospital course

The patient was managed in a monitored inpatient setting with decongestive therapy and guideline-directed heart failure pharmacotherapy titrated according to hemodynamic tolerance, alongside targeted correction of metabolic derangements. Treatment focused on simultaneous stabilization of heart failure physiology and correction of profound hypocalcemia/hypomagnesemia with vitamin D replacement (including active vitamin D), with biochemical monitoring and structured cardiology-endocrinology follow-up (Table 2). However, the independent contribution of guideline-directed medical therapy versus metabolic correction to recovery cannot be definitively separated.

**Table 2:** Discharge management plan MR: modified-release; CoQ10: coenzyme Q10; µg: microgram; IU: international units

Domain	Therapy/Plan
Heart failure therapy	Sacubitril/valsartan 25 mg twice daily; bisoprolol 1.25 mg once daily; ivabradine 2.5 mg twice daily; torsemide 5 mg once daily; spironolactone 12.5 mg once daily
Symptom/adjunct	Trimetazidine MR 35 mg twice daily; ubiquinone/CoQ10 supplement
Calcium, vitamin D, and magnesium correction	Oral calcium supplementation (two preparations); calcitriol 0.25 µg twice daily; cholecalciferol 60,000 IU weekly for six weeks; magnesium hydroxide tablets (each providing ~100 mg elemental magnesium) one tablet twice daily with food
Follow-up	Cardiology outpatient review with renal function testing; endocrinology follow-up with repeat serum calcium and albumin within one month of discharge

Clinical pharmacy note

Calcium and vitamin D supplementation were administered with counseling regarding medication timing, potential drug-nutrient interactions, and administration of vitamin D with meals to optimize absorption. Oral magnesium replacement (magnesium hydroxide providing ~100 mg elemental magnesium twice daily with food) was included to correct hypomagnesemia and support parathyroid hormone (PTH)-mediated calcium normalization; this formulation was used because it was locally available and well tolerated, although organic magnesium salts may have greater bioavailability. Discharge counseling included medication adherence education and monitoring for potential adverse effects because of the substantial treatment burden.

Follow-up and outcome

Baseline echocardiography documented severe left ventricular systolic dysfunction with an EF of 35% and severe functional mitral/tricuspid regurgitation (MR/TR). Serial follow-up echocardiography demonstrated progressive improvement in systolic function with EF 40% at the one-month follow-up and 55% at the three-month follow-up, accompanied by symptomatic improvement. At the one-year follow-up, 2D echocardiography with color Doppler documented normal biventricular function with EF 65% and fractional shortening 36%, LV dimensions end-diastolic diameter 4.5 cm and end-systolic diameter 2.8 cm, no regional wall motion abnormality, no pericardial effusion, and no mitral, tricuspid, aortic, or pulmonary regurgitation, confirming recovery of systolic function and resolution of previously documented functional MR/TR secondary to LV dilatation.

Follow-up intervals at one month, three months, and one year were selected to document early response after metabolic correction, intermediate reverse remodeling, and durability of recovery (Table 3).

**Table 3:** Serial echocardiographic recovery of LV function EF: ejection fraction; FS: fractional shortening; MR: mitral regurgitation; TR: tricuspid regurgitation; AR: aortic regurgitation; PR: pulmonary regurgitation; LV: left ventricle; EDD: end-diastolic diameter; ESD: end-systolic diameter.

Time point	EF (%)	MR/TR	Comment
Baseline	35	Severe MR/TR (functional)	Severe LV dysfunction
1 month	40	Improving	Symptomatic improvement
3 months	55	Further improved	Recovery trajectory
1 year	65 (FS 36%)	No MR/TR/AR/PR	Normal biventricular function; LV EDD 4.5 cm, ESD 2.8 cm

**Figure 2:**
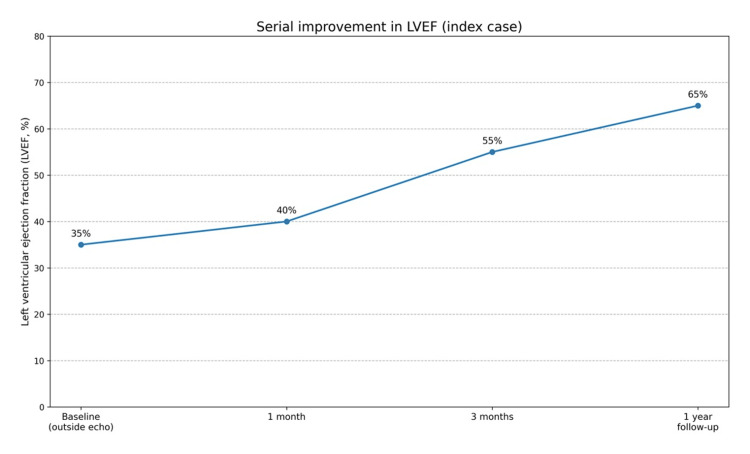
Serial improvement in left ventricular ejection fraction (LVEF) in the index case. Trend showing progressive improvement in LVEF from baseline (35%) to one month (40%) and three months (55%), with echocardiography at the one-year follow-up demonstrating normalization (65%) and resolution of valvular regurgitation.

Serial improvement in LVEF over follow-up is summarized in Figure 2.

## Discussion

This case demonstrates a rare but clinically important principle: severe LV systolic dysfunction in a young patient may be metabolically mediated or strongly associated with reversible metabolic derangements, and recognition may meaningfully alter prognosis. Although vitamin D deficiency is common, vitamin D deficiency-associated hypocalcemic cardiomyopathy remains uncommon in published literature and can be missed when symptoms are attributed to primary dilated cardiomyopathy or presumed myocarditis. A pediatric review emphasized hypocalcemic rachitic cardiomyopathy as a serious but treatable complication of deficiency states and highlighted recovery when diagnosis and correction occur early [1]. Adult and pediatric reports also support improvement of LV function after correcting hypocalcemia with vitamin D therapy [2,3]. The present report is clinically instructive because it combines a coherent endocrine signature (profound total and ionized hypocalcemia with markedly elevated PTH and low 25-hydroxyvitamin D), a severe clinical phenotype with functional MR/TR, and a serial EF recovery trajectory culminating in normalization at the one-year follow-up.

Why now? Possible drivers of vitamin D deficiency in an adolescent

In adolescents, low vitamin D status may reflect low dietary vitamin D and calcium intake, reduced sunlight exposure (lifestyle and geography), higher skin melanin content, and increased growth-related demands; less commonly, malabsorption (e.g., celiac disease), chronic liver disease, chronic kidney disease, or medication exposures affecting vitamin D metabolism can contribute. In the available record, a detailed dietary/sun exposure history and formal evaluation for malabsorption were not documented. This uncertainty does not weaken the central clinical message-LV function improved in parallel with correction of the mineral/vitamin D axis-but it should be acknowledged transparently.

The novelty of this report is not vitamin D deficiency alone, but the integrated phenotype and objective trajectory: adolescent presentation with documented ionized hypocalcemia and marked secondary hyperparathyroidism, severe functional MR/TR during decompensation, and a multi-timepoint echocardiographic recovery pattern culminating in normal biventricular function with complete resolution of valvular regurgitation at the one-year follow-up. This combination strengthens causal plausibility beyond a single timepoint association and provides a practical “don’t miss” diagnostic and management message, although causality remains inferential due to concurrent therapeutic interventions.

Given this trajectory, the mechanistic link between disordered mineral metabolism and contractile dysfunction is clinically relevant to highlight.

Pathophysiology and the role of magnesium

The mechanistic association between hypocalcemia and myocardial dysfunction is biologically strong. During the ventricular action potential plateau (phase 2), calcium influx through L-type calcium channels provides the trigger for calcium-induced calcium release from the sarcoplasmic reticulum via ryanodine receptors, generating the intracellular calcium transient required for actin-myosin cross-bridge cycling and contraction [6]. When extracellular calcium is profoundly reduced, the trigger calcium current may be blunted, reducing sarcoplasmic reticulum calcium release and diminishing contractile force, manifesting as systolic failure [6]. Vitamin D deficiency contributes through reduced intestinal calcium absorption and compensatory secondary hyperparathyroidism; standardized diagnostic thresholds and treatment strategies are described in endocrine guidance [7]. Concomitant hypomagnesemia can perpetuate hypocalcemia through impaired PTH secretion and/or PTH resistance; classic physiology supports reduced PTH secretion in magnesium deficiency, explaining why magnesium correction may be required for durable calcium normalization and recovery [8]. However, these mechanisms explain plausibility but do not establish the exclusivity of causation in this clinical case.

CMR relevance and diagnostic framing

Myocarditis remains a competing diagnosis in young patients with severe LV dysfunction, particularly when cardiac biomarkers are abnormal. CMR can strengthen diagnostic confidence by assessing ventricular function and, when tissue characterization is available, evaluating inflammatory overlap. A pediatric cohort evaluating hypocalcemia-induced dilated cardiomyopathy highlighted that CMR may assist prognostication and help identify myocarditis overlap in selected cases [4]. In the present case, cine imaging supported the severity of systolic dysfunction during decompensation, while serial echocardiography documented progressive recovery and later normalization, supporting a reversible metabolic phenotype in the differential diagnosis context.

Other differentials considered in a young patient with severe LV dysfunction included myocarditis, primary dilated cardiomyopathy, and endocrine/metabolic causes. The coherent mineral metabolism abnormalities and the serial recovery of EF with resolution of functional MR/TR support a likely metabolic contribution and a reversible phenotype in this case.

International comparison and context

International reports consistently describe improvement in LV systolic function after correction of hypocalcemia with calcium and vitamin D replacement [2,3]. A pediatric cohort further emphasizes that outcomes are generally favorable when hypocalcemia is promptly corrected and that CMR may help identify myocarditis overlap and refine prognosis in selected cases [4]. Importantly, reversibility is not universal; severity at presentation and diagnostic delay may influence recovery, highlighting the value of early screening and structured follow-up [9]. Adult cases of reversible cardiomyopathy and severe congestive heart failure due to hypocalcemia further support this mechanism across age groups [10,11].

Table 4 benchmarks the present case against selected international reports and cohorts.

**Table 4:** International comparison highlighting clinical value of the present case EF: ejection fraction; LV: left ventricle; DCM: dilated cardiomyopathy; MR: mitral regurgitation; TR: tricuspid regurgitation; NYHA: New York Heart Association; CMR: cardiac magnetic resonance; iCa: ionized calcium; Ca: calcium; PTH: parathyroid hormone; 25(OH)D: 25-hydroxyvitamin D; NT-proBNP: N-terminal pro–B-type natriuretic peptide; NR: not reported; Vit D: vitamin D

Study (Year)	Patient group	Trigger / biochemical pattern	Cardiac severity at presentation	Treatment focus	Recovery/follow-up	What the present case adds
Present case	Adolescent (13/F)	Vit D deficiency + profound hypocalcemia (Ca 6.1; iCa 0.79) + secondary hyperPTH (PTH 658) + hypomagnesemia (Mg 1.5)	Dilated cardiomyopathy phenotype with severe LV dysfunction + severe functional MR/TR; NYHA III; NT-proBNP 13,200	Guideline-directed medical therapy + decongestion + Ca/Mg/Vit D correction	Serial EF recovery 35→40→55→65%; MR/TR resolved; normal biventricular function on later echo	Adolescent phenotype + severe functional MR/TR + ionized Ca documented + strong endocrine coherence + multi-timepoint objective recovery to normal EF
Elidrissy et al., 2013 [1]	Pediatric review	Hypocalcemic rachitic cardiomyopathy (deficiency states)	Severe heart dailure reported across cases	Ca + Vit D correction emphasized	Recovery often reported when treated early	Supports rarity/importance; frames “don’t miss” metabolic cause
Sung et al., 2010 [2]	Adult	Hypocalcemia (post-thyroidectomy)	DCM with reduced EF	Ca + Vit D replacement	EF improved and regurgitation improved over follow-up	Confirms reversibility exists across etiologies; adult comparator
Kim et al., 2010 [3]	Infant	Vit D deficiency rickets (hypocalcemia)	Severe LV dysfunction	Ca + Vit D	Marked EF recovery on follow-up	Classic infant phenotype: case extends to adolescence + MR/TR + ionized Ca
Garg et al., 2021 [4]	Pediatric cohort	Hypocalcemia-induced DCM (variable Vit D/PTH)	Mean EF ~20% at admission	Correction + imaging-guided assessment	Mean EF improved at discharge and longer follow-up; CMR prognostic value	Cohort context + CMR role; this case adds valve phenotype + long-interval normal echo
Fabi et al., 2013 [9]	Pediatric case	Hypocalcemic rickets	DCM	Ca + Vit D	Reversibility not universal; depends on the severity/delay	Supports why documenting recovery trajectory and timing matters
Avsar et al., 2004 [10]	Adult case	Hypocalcemia	Reversible DCM	Correction	Recovery documented	Additional adult evidence supporting reversibility
Solzbach et al., 2010 [11]	Adult case	Severe hypocalcemia	Severe congestive heart failure	Correction	Recovery documented	Reinforces severity can still be reversed with treatment

Compared with prior reports that predominantly involve infants or adult post-surgical hypocalcemia, the present case is notable for an adolescent phenotype with documented ionized hypocalcemia, marked secondary hyperparathyroidism, severe functional MR/TR, and a multi-timepoint EF recovery trajectory to normalization, thereby strengthening causal plausibility and clinical teachability.

Limitations

Baseline and early follow-up echocardiography images were not available for inclusion; however, EF values were documented in serial echocardiography reports and are supported by echocardiography at the one-year follow-up, demonstrating normalization of LV function and resolution of regurgitation. Evaluation for the underlying etiology of vitamin D deficiency (dietary intake, sun exposure, and malabsorption, such as celiac disease) was not fully documented in the available records. The contribution of concurrent guideline-directed heart failure therapy to ventricular recovery cannot be isolated from metabolic correction effects.

Learnings

Hypocalcemia is a rare but clinically important and potentially reversible contributor within a broader metabolic context of heart failure in young patients presenting with acute LV systolic dysfunction; it should be actively considered in evaluation. A structured screen for total calcium, ionized calcium, magnesium, phosphate, PTH, and 25-hydroxyvitamin D should be obtained early in severe or disproportionate LV dysfunction. Profound hypocalcemia can depress contractility by blunting L-type calcium channel-mediated trigger calcium influx and sarcoplasmic reticulum calcium release, reducing the systolic calcium transient [6]. Hypomagnesemia can perpetuate hypocalcemia through impaired PTH secretion/action; magnesium assessment and correction are essential in this phenotype [8].

## Conclusions

Vitamin D deficiency-associated hypocalcemia with secondary hyperparathyroidism is an uncommon but treatable contributor to severe LV dysfunction. In this adolescent, correction of mineral and vitamin D abnormalities alongside heart failure therapy was followed by serial improvement and eventual normalization of systolic function (EF 65%) with resolution of functional MR/TR secondary to LV remodeling. Early electrolyte-endocrine screening should be integrated into the evaluation of severe LV dysfunction in young patients to identify reversible etiologies and improve outcomes.
